# Hospital admissions during Covid-19 lock-down in Germany: Differences in discretionary and unavoidable cardiovascular events

**DOI:** 10.1371/journal.pone.0242653

**Published:** 2020-11-20

**Authors:** Elisabeth Stöhr, Adem Aksoy, Meghan Campbell, Muntadher Al Zaidi, Can Öztürk, Julia Vorloeper, Jonas Lange, Atsushi Sugiura, Nihal Wilde, Marc Ulrich Becher, Christian Diepenseifen, Ulrich Heister, Georg Nickenig, Sebastian Zimmer, Vedat Tiyerili

**Affiliations:** 1 Department of Cardiology, Heart Center, University of Bonn, Bonn, Germany; 2 Institute of Clinical Chemistry & Clinical Pharmacology, University of Bonn, Bonn, Germany; 3 Amt für Bevölkerungsschutz, Siegburg, Germany; 4 Department of Anesthesiology and Critical Care Medicine, University of Bonn, Bonn, Germany; Azienda Ospedaliero Universitaria Careggi, ITALY

## Abstract

**Background:**

A decline in hospitalization for cardiovascular events and catheter laboratory activation was reported for the United States and Italy during the initial stage of the Covid-19 pandemic of 2020. We report on the deployment of emergency services for cardiovascular events in a defined region in western Germany during the government-imposed lock-down period.

**Methods:**

We examined 5799 consecutive patients who were treated by emergency services for cardiovascular events during the Covid-19 pandemic (January 1 to April 30, 2020), and compared those to the corresponding time frame in 2019. Examining the emergency physicians’ records provided by nine locations in the area, we found a 20% overall decline in cardiovascular admissions.

**Results:**

The greatest reduction could be seen immediately following the government-imposed social restrictions. This reduction was mainly driven by a reduction in discretionary admissions for dizziness/syncope (-53%), heart failure (-38%), exacerbated COPD (-28%) and unstable angina (-23%), while unavoidable admissions for ST-elevation myocardial infarction (STEMI), cardiopulmonary resuscitation (CPR) and stroke were unchanged. There was a greater decline in emergency admissions for patients ≥60 years. There was also a greater reduction in emergency admissions for those living in urban areas compared to suburban areas.

**Conclusions:**

During the Covid-19 pandemic, a significant decline in hospitalization for cardiovascular events was observed during the government-enforced shutdown in a predefined area in western Germany. This reduction in admissions was mainly driven by “discretionary” cardiovascular events (unstable angina, heart failure, exacerbated COPD and dizziness/syncope), but events in which admission was unavoidable (CPR, STEMI and stroke) did not change.

## Introduction

In December 2019, a new viral pneumonia was detected in Wuhan, China [1]. The clinical manifestation, named coronavirus disease 2019 (Covid-19), is caused by the recently identified severe acute respiratory syndrome coronavirus 2 (SARS-CoV-2) [2]. Covid-19 quickly spread to become a global pandemic with currently around 9 million validated infections and >470,000 casualties (www.who.org). Over the last months, most countries have imposed unprecedented public restrictions and social-distancing rules to contain the outbreak and lessen the demand on the health care systems.

Depending on the timing of these restrictions and the public’s adherence to them, the results have been mixed. In some areas, where restrictions were imposed later or less stringently, the infection rate grew rapidly and hospitals became overwhelmed, just treating the most severe cases of Covid-19. Particularly in Wuhan, northern Italy, Madrid, the Alsace area of France, New York City and Brazil, hospitals were working beyond their capacity. The intense media coverage of these events, including images of bodies accumulating in the morgues, doctors attending to patients without face masks and warning signs on hospital entrances about the virus, produced enormous fear in the general public.

In Germany, the situation started out quite differently. Strict guidelines on social distancing, closed schools and universities, and travel restrictions were announced by the federal government on March 13, 2020 and continued until April 30, 2020. This, in combination with an intense effort to track infection chains and test for the virus, kept the rate of Covid-19 low enough to avoid the overburdened hospitals that were seen elsewhere. In fact, during this time most non-essential surgeries and hospital visits were cancelled to free up resources for the anticipated Covid-19 patients, leaving many hospitals with ample free beds.

Although these measures turned out to be effective in curbing the virus for some time, German citizens were still exposed to media images from the harder-hit areas and warned by the government to avoid unnecessary doctor and hospital visits. The result was a high level of fear, especially amongst older residents, who are at an increased risk for both Covid-19 and cardiovascular events.

While recent studies from areas that were directly impacted by an overwhelming Covid-19 burden found a decrease in acute myocardial infarctions [3, 4] and a decline in activation of catheter laboratories [5], it remains unclear if these findings are the result of patients refraining their behaviour during the lock-down period or due to the limited availability of health care services. Thus, in our study we aimed to evaluate the deployment of emergency services and consecutive hospital admissions in a predefined region and time period where the medical infrastructure had not yet reached its capacity. We also studied differences in the type of cardiovascular event presented, as well as the age, gender and location of the patient admitted. This study is focused on the effects of the initial lock-down period in Germany, however, with newly skyrocketing rates of Corona virus infection and the next round of government-imposed restrictions imminent, this information is more important now than ever.

## Methods

All emergency physicians’ records containing the preclinical diagnosis of unstable angina pectoris, ST-elevation myocardial infarction (STEMI), heart failure (HF), uncontrolled hypertension, arrhythmic event, cardiopulmonary resuscitation (CPR), syncope/dizziness and stroke were included. The prospectively assessed records were obtained from January 1 to April 30, 2020 while records of the corresponding dates from 2019 made up the control period. February 29, 2020 was removed from the analysis to keep the overall number of days the same, because 2020 was a leap year. The obtained records included date, alarm time, name, date of birth, address, brief summary of the acute symptoms, preclinical diagnosis, vital parameters, drugs administered during the preclinical treatment and destination hospital. These records were obtained from nine consecutive emergency departments in western Germany. Two of these departments were in Bonn and seven were in Rhein-Sieg- Kreis. Overall these departments cover emergency deployments for roughly one million residents.

The study protocol was approved by the ethics committee of the Medical Faculty of the Rheinische Friedrich-Wilhelms-Universität Bonn (approval number: AZ 177/20). The requirement for informed consent was waived because the data were analysed anonymously.

### Statistics

Characteristics of the study patients are described as mean values (with standard deviation (sd)) or numbers with percentages as appropriate. Admissions were analysed as response variables individually and by grouping them into "discretionary" (Unstable angina, HF, COPD, Hypertension, Arrhythmia, Dizziness/Syncope) and "unavoidable" (STEMI, CPR, stroke)

Daily hospital admission curves are depicted as mean values with smoothed curves ± four days and include ± one standard deviation. Differences in the response variables between 2020 and 2019 were assessed using Poisson regression models. Subgroup analyses of the number of “discretionary” and “unavoidable” admissions were carried out for gender, age (≥60y, <60y) and living area (urban, suburban). Estimates obtained from the models are shown with 95% confidence intervals (CI).

The Bonferroni correction was applied to adjust for multiple comparisons. Statistical analysis was performed with SPSS v 25 and R Software for Statistical Computing Version 3.6.1.

## Results

We included 5799 patients from nine emergency departments in western Germany between January 1 and April 30, 2020 and the corresponding control period in 2019. The average age of all patients was 71.9 ± 15.1 years and 2916 (50.3%) were female. The primary symptom for emergency dispatch was unstable angina pectoris in 1442 (24.9%), followed by uncontrolled arterial hypertension in 1047 (18.1%), stroke in 956 (16.5%), exacerbated chronic obstructive pulmonary disease (COPD) in 664 (11.5%), arrhythmia in 758 (13.1%), decompensated heart failure (HF) in 489 (8.4%), dizziness/syncope in 429 (7.4%), ST-elevation myocardial infarction (STEMI) in 286 (4.9%) and cardiopulmonary resuscitation in 239 (4.1%) (percentages add to >100% due to multiple diagnoses in some patients).

**Fig 1:**
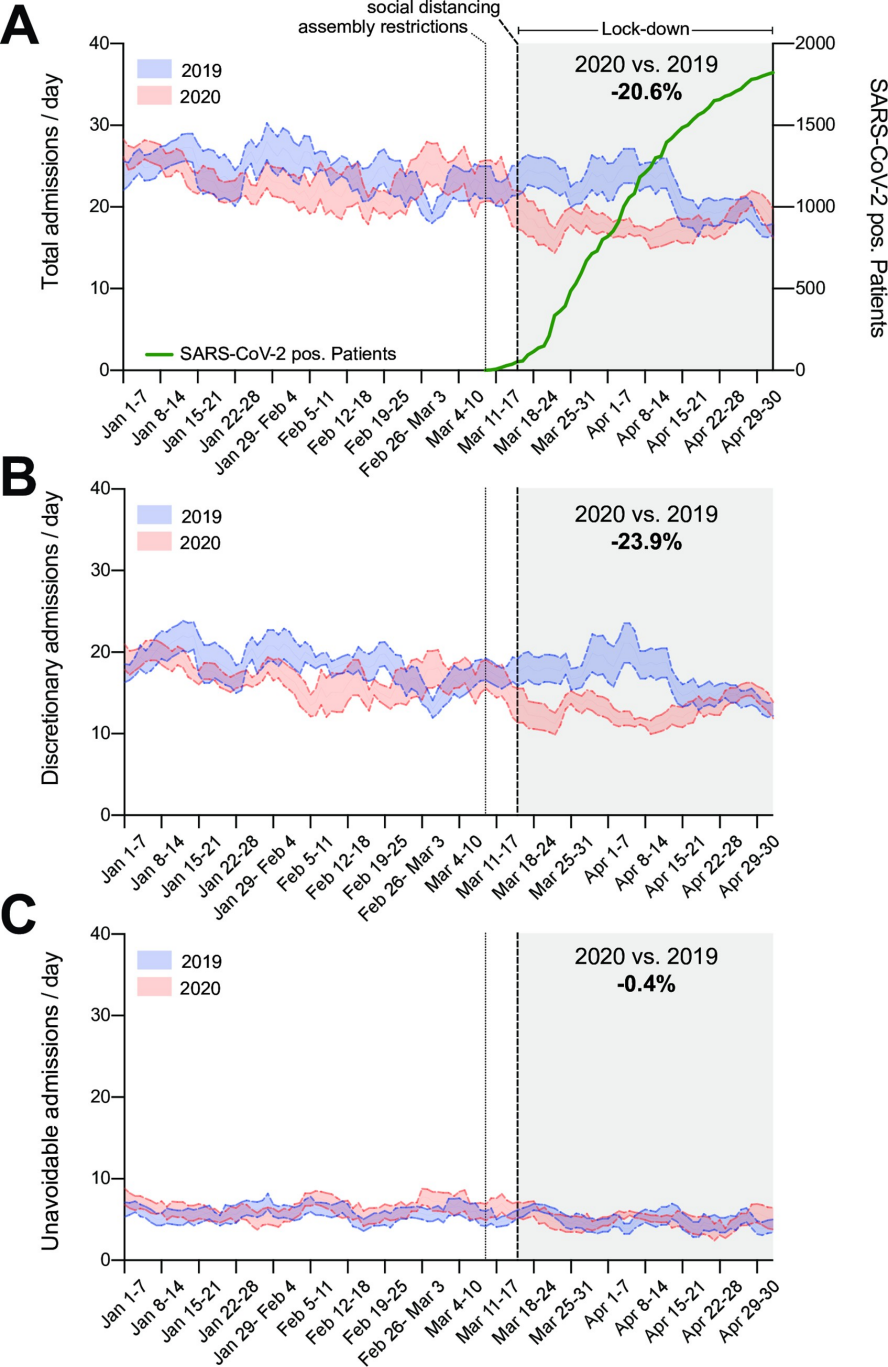
Comparison of hospital admission rates due to cardiovascular diagnoses between 2019 and 2020. A) Shown are total hospital admissions per day for January 1 through April 30. Righthand y-axis depicts the cumulative SARS-CoV-2 patients for the studied region. B) Shown are discretionary hospital admissions per day for January 1 through April 30. C) Shown are unavoidable hospital admissions per day for January 1 through April 30. (A-C) The mean using curve smoothing ± one standard deviation.

The first validated Covid-19 patient in Germany was publicly announced at the end of January, while the first positive SARS-CoV-2 infection in the examined area occurred on February 28. Shortly after, an outbreak during a Carnival-associated festivity with more than 100 new cases ensued a lock-down of the region with assembly restrictions (Fig 1A). On March 13, the German government announced strict social-distancing measures, including the closing of kindergartens, schools, retail stores, restaurants and all non-essential businesses, and a shelter-in-place order was issued (Fig 1A).

We analysed the incidence of hospital admissions for cardiovascular disease via paramedic and emergency physician reports. Between January 1 and the start of the lock-down on March 13 no differences in emergency hospital admissions could be registered (Fig 1A, S1 Table). As of the third week in March, coinciding with the government-imposed measures for social containment, a marked reduction of 20% could be seen.

Some cardiovascular events, including STEMI, cardiac arrest and stroke, are independent of the patient’s assessment of the risk/benefit balance for seeking medical attention (risk of suffering from cardiovascular disease vs. contracting the virus) and ultimately result in “unavoidable admission” to a hospital. We inquired whether the observed decline in cardiovascular admissions during lock-down similarly affected these events. When the cohort was split into “discretionary” and “unavoidable” admissions (Fig 1B and 1C), we found the incidence of unavoidable admission was stable throughout the entire period (Fig 1C), while discretionary admission rates dropped during the lock-down by 23.9% (Fig 1B).

**Fig 2:**
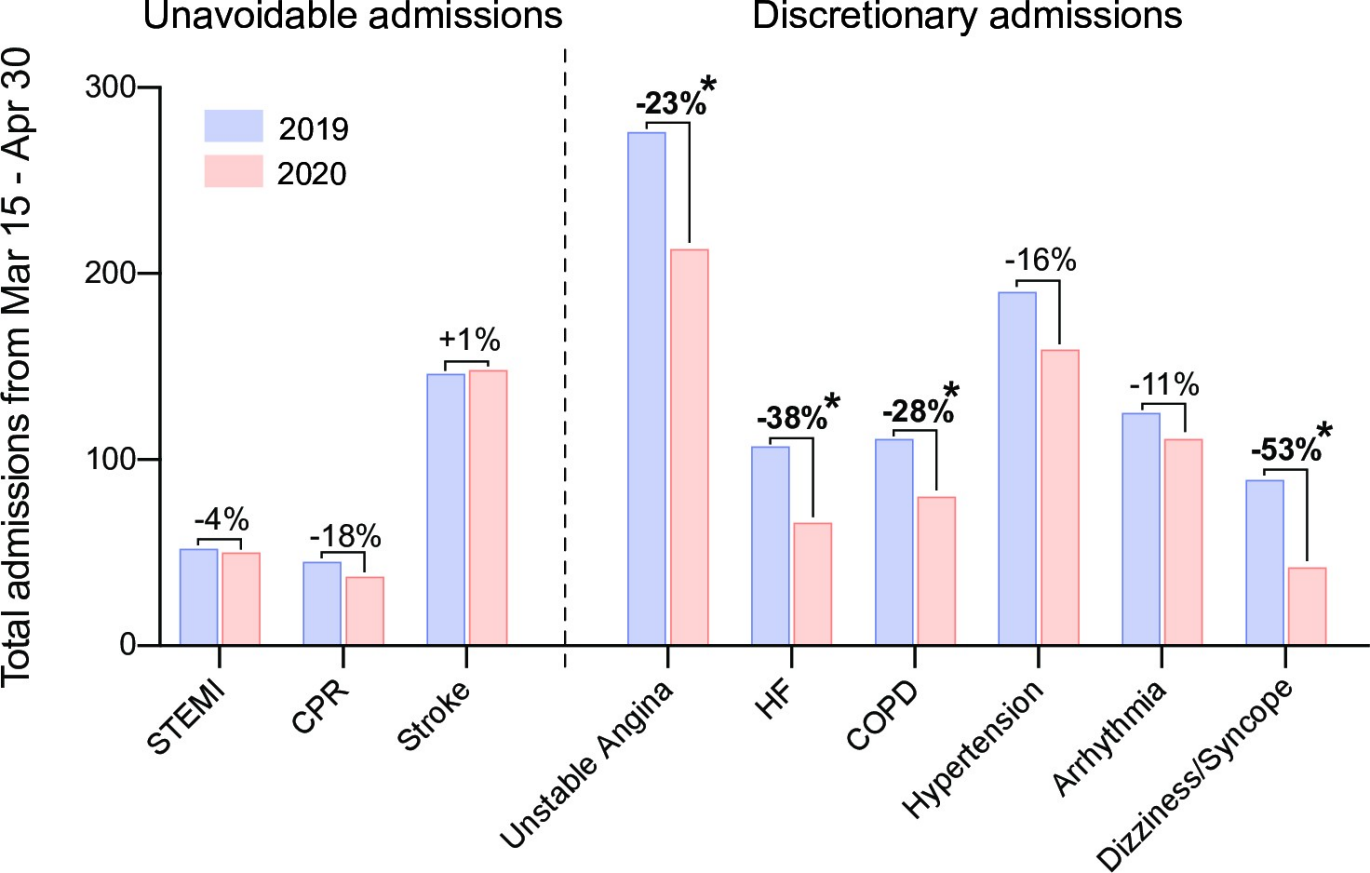
Analysis of total admissions according to diagnoses during the lock-down period. Numbers were collected from March 15 to April 30 from 2019 and 2020. Bold label and asterisk indicate a significant difference between 2020 and 2019 in the Poisson Model. (2020 vs. 2019 for unstable angina p = 0.004, HF p = 0.002, COPD p = 0.033, dizziness/syncope p < 0.001). STEMI, ST-elevation myocardial infarction; CPR, cardiopulmonary resuscitation; HF, decompensated heart failure; COPD, exacerbated chronic obstructive pulmonary disease.

Amongst the unavoidable admissions, stroke was most prevalent followed by STEMI and CPR (Fig 2). There was no significant difference between 2019 and 2020 for any of these diagnoses (S2 Table). On average, the rate of discretionary admissions was 23.9% lower during 2020. The largest contributors to this effect were unstable angina and hypertension, while the largest relative decreases were seen with dizziness/syncope and heart failure (S2 Table).

**Fig 3:**
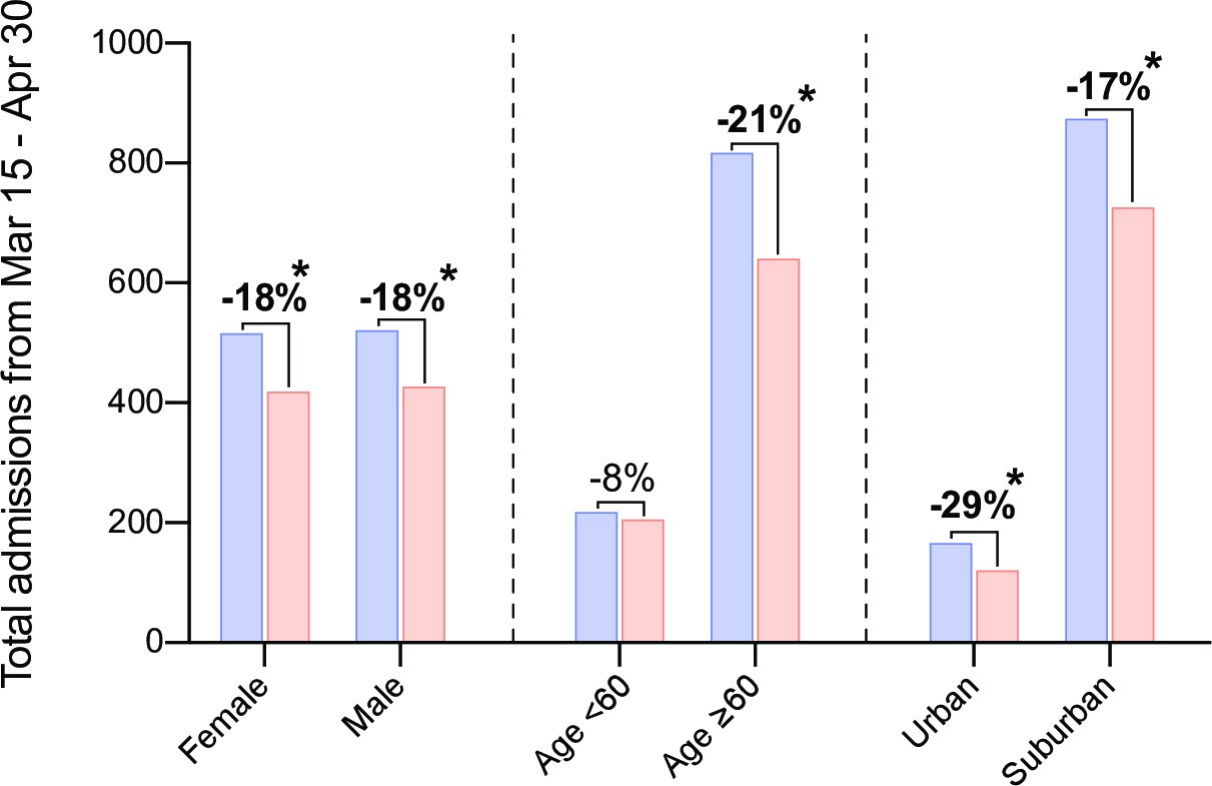
Analysis of total admissions according to patient characteristics during the lock-down period. Numbers were collected from March 15 to April 30 from 2019 and 2020. Bold label and asterisk indicate a significant difference between 2020 and 2019 in the Poisson Model. (2020 vs. 2019 for female p = 0.006, male p = 0.003, age≥60 p < 0.001, suburban p = 0.001, urban p = 0.015).

Next, we analysed patient characteristics with respect to the rate of hospital admissions. We found an equal, but significant decrease in hospital admissions in both female and male patients (S3 Table). People 60 years or older were shown to be at a higher risk for developing a complicated and severe course of Covid-19 and therefore were told to be extremely cautious in their social interactions. (https://www.rki.de/DE/Content/InfAZ/N/Neuartiges_Coronavirus/Steckbrief.html). This population of patients also displayed a significant decline in overall admissions due to cardiovascular events during the lock-down period (Fig 3, S3 Table). Whereas, the younger patient population (<60 years) showed no significant change in the use of emergency services.

Finally, we conducted a comparison between residents of Bonn, a city with around 400,000 inhabitants and more than 3600 inhabitants per km^2^ (https://www2.bonn.de/statistik/dl/ews/Bevölkerungsstatistik_2017.pdf), and Rhein-Sieg-Kreis with 600,000 inhabitants and an average population density of 520 inhabitants per km^2^ (https://www.rhein-sieg-kreis.de/verwaltung-politik/kreis/wissenwertes-zum-rsk/c_der-rhein-sieg-kreis-in-zahlen.php). There was an association between location and decrease in emergency admissions, as the rate went down more steeply in the urban compared to the suburban area (Fig 3, S3 Table).

## Discussion

The key findings of this study are

1. A substantial reduction in discretionary admissions at the beginning of the social-distancing regulations
2. Stable rate of unavoidable admissions (CPR, STEMI and stroke) throughout the lock-down
3. Reduction of admissions for patients 60 years and older and from urban areas

With this study, we aimed to generate a comprehensive but local representation of a population during the Covid-19 pandemic that had not changed significantly since the corresponding control period in the previous year, to prevent confounding influences on the occurrence of the cardiovascular events of interest. In the area we studied, compared to other populations that have been studied for cardiovascular events during the Covid-19 pandemic, SARS-CoV-2 was not as prevalent and the health-care system has thus far remained below capacity. Furthermore, we did not see a relevant reduction of the non-negotiable or unavoidable preclinical diagnoses in a composite end-point of STEMI, stroke and CPR.

A recently published study from northern Italy found a significant reduction in admissions for all acute coronary syndromes, including unstable angina, NSTEMI and STEMI, between March 12 and 19, 2020 [3]. Furthermore, those patients who were admitted developed significantly more complications and treatment in the cardiac catheterization lab for these entities was delayed. A similar study in Hong Kong also found significant delays in the care of STEMI patients during the SARS-CoV-2 outbreak [6]. In contrast to our study, this phenomenon can likely be attributed to the physicians and emergency rooms already being saturated with critical Covid-19 patients and/or increased viral containment measurements.

Garcia et al. found a 38% reduction in activation of cardiac catheterization laboratories for STEMI during the Covid-19 pandemic (from March 1 to 31) in nine high-volume centres in the United States [5]. Notably, this study focused only on STEMI patients and did not analyse the other forms of cardiovascular events described by our study. One additional factor that could have contributed to this result is that many U.S. citizens lost their jobs and their health insurance during the pandemic. This could be a confounding factor in the patient’s decision to seek medical attention. In contrast, German residents are guaranteed to receive paid health care by the federal government, preventing concern about the financial aspects of a visit to the hospital.

While we found a similar reduction of around 20% for discretionary cardiovascular admissions during the Covid-19 lock-down period, the rate of unavoidable admissions remained unchanged. These findings are in line with a report from a single emergency department at the University Hospital Ulm (Germany) with a cohort of 94 patients [7]. They saw a reduction in patients presenting with NSTEMI but not other forms of cardiovascular events, including STEMI and out of hospital cardiac arrest. In our analyses, unstable angina was reduced by 23% during the lockdown period when compared with the same time period of the previous year. In contrast, De Filippo et al. reported a similar incidence of unstable angina during the Corona virus outbreak as to the previous year. These different results may be for three different reasons. First of all, the definition of the study period was based on different settings. We defined our study term to be during the government-issued lockdown period in Germany, not depending on the incidence of Covid-19. In contrast, De Felippo et al. defined their study period beginning with the first confirmed case of Covid-19 in Italy (February 20, 2020), which is independent of any governmental decisions and in Italy the stricter governmental restrictions did not come until the virus was already well established in the North. Second, the rate of Covid-19 and overburdened hospitals were reported to be very high there, whereas here in Germany we had a low rate of Covid-19 in comparison. Third, the sample sizes differed greatly (n = 5799 for our study vs. n = 2202 De Felippo et al.) between the two studies.

There are several possible explanations for a change in hospital admissions during a pandemic. In considering the conditions of our study population, that is, residing in an area where there is great social awareness and unease about the virus but not a high incidence rate or overburdened medical system, we hypothesize that the decrease in hospital admissions due to “discretionary” cardiac events is largely due to fear of becoming infected at the hospital.

In contrast, one could argue that during high-stress periods, such as a viral pandemic, there would be even more cardiovascular events triggered. Stress has clearly been linked to the incidence of cardiovascular disease [8], including both chronic stress and acute stress, as was documented during the 2006 World Cup matches in Germany [9] and the Los Angeles earthquake of 1994 [10]. The current pandemic situation can probably be classified somewhere between a chronic and acute stressor, but there is no doubt that stress levels have increased for many people during this time. In addition to the fear of potentially becoming ill, many people also experienced added stress due to home-schooling their children, loss of a job, or social isolation. Therefore, it could be reasonably expected that the admission for cardiovascular events during this time would have actually increased, but instead we observed the opposite effects.

For older people, there was an added concern and stress about being infected by SARS-CoV-2. There is a clear association between advanced age and the seriousness of Covid-19 [11]. As this fact was widely published by the media, it likely hampered older people to leave their home during the lock-down period. We found a more significant decrease in hospital admissions for cardiovascular causes by people 60 years and older. For this population, the fear of contracting the virus probably outweighed their concerns due to their cardiac symptoms.

We found that patients living in an urban setting were more likely to avoid seeking cardiac emergency services than those living in less densely populated areas. We speculate that suburban residents feel more secure in their surroundings and perceive a lower risk of infection when seeking medical attention compared to people living in more crowded urban neighbourhoods.

The fact that the rate of cardiac admissions of all types normalized after the first three weeks of the lock-down could possibly be due to lowered concerns about getting infected and increasing severity of formerly ignored symptoms. It will be interesting to see if this pattern is repeated now as we enter the next phase of governmental restrictions on public life due to increasing rates of Corona virus in the general population.

### Study limitations

Because this is an observational study, true causality for the results cannot be determined. The population studied within this area is relatively homogeneous and therefore, may not be representative of the entire population. We studied a defined time period within the course of the pandemic, and the results generated herein may not be applicable to a setting of much lower viral infection and fear. In addition, we were not able to identify the people who did not alert emergency services due to cardiovascular systems, so this data could not be included in our assessment. It is also possible that there were some other factors that were not controlled for in our study (e.g. weather, holidays, etc.). The out-of-hospital mortality rate has not been analysed here. Unfortunately, this data is not yet available in Germany, although this information would be highly valuable for regulatory bodies and government officials when evaluating and implementing social-distancing regulations.

## Conclusions

Our study found a reduction of hospital admissions for discretionary cardiovascular events during government-imposed restrictions for the Covid-19 pandemic in Germany, while the rate of unavoidable admissions remained the same. We found an unambiguous association between the onset of the shelter-in-place order by the German government and the reduction of hospital admissions. In particular, the decrease was greater in elderly patients and those living in an urban setting. This study informs about the effects of wide-scale social restrictions on the performance of the health care system, independent of the particular viral disease being combated.

## Supporting information

S1 Table Statistical calculations for Fig 1: March 15-April 30. (DOCX)

S2 Table Statistical calculations for Fig 2: March 15-April 30. (DOCX)

S3 Table Statistical calculations for Fig 3: March 15-April 30. (DOCX)

## References

[1] Huang C, Wang Y, Li X, et al Clinical features of patients infected with 2019 novel coronavirus in Wuhan, China. The Lancet 2020;395:497–506. 10.1016/S0140-6736(20)30183-5 PMC7159299 31986264

[2] Zhou P, Yang X-L, Wang X-G, et al A pneumonia outbreak associated with a new coronavirus of probable bat origin. Nature 2020;579:270–273. 10.1038/s41586-020-2012-7 32015507 PMC7095418

[3] Rosa SD, Spaccarotella C, Basso C, et al Reduction of hospitalizations for myocardial infarction in Italy in the COVID-19 era.:6. 10.1093/eurheartj/ehaa409 PMC7239145 32412631

[4] De Filippo O D’Ascenzo F, Angelini F, et al Reduced Rate of Hospital Admissions for ACS during Covid-19 Outbreak in Northern Italy. N Engl J Med 2020:NEJMc2009166. 10.1056/NEJMc2009166 32343497 PMC7224608

[5] Garcia S, Albaghdadi MS, Meraj PM, et al Reduction in ST-Segment Elevation Cardiac Catheterization Laboratory Activations in the United States During COVID-19 Pandemic. Journal of the American College of Cardiology 2020;75:2871–2872. 10.1016/j.jacc.2020.04.011 32283124 PMC7151384

[6] Tam C-CF, Cheung K-S, Lam S, et al Impact of Coronavirus Disease 2019 (COVID-19) Outbreak on ST-Segment–Elevation Myocardial Infarction Care in Hong Kong, China. Circ: Cardiovascular Quality and Outcomes 2020;13 Available at: https://www.ahajournals.org/doi/10.1161/CIRCOUTCOMES.120.006631. Accessed June 23, 2020. 10.1161/CIRCOUTCOMES.120.006631 PMC7147280 32182131

[7] Rattka M, Baumhardt M, Dreyhaupt J, et al 31 days of COVID-19—cardiac events during restriction of public life—a comparative study. Clin Res Cardiol 2020 Available at: http://link.springer.com/10.1007/s00392-020-01681-2. Accessed June 23, 2020. 10.1007/s00392-020-01681-2 32494921 PMC7268583

[8] Dimsdale JE. Psychological Stress and Cardiovascular Disease. Journal of the American College of Cardiology 2008;51:1237–1246. 10.1016/j.jacc.2007.12.024 18371552 PMC2633295

[9] Ute W-L, David L, Sonja G, et al Cardiovascular Events during World Cup Soccer. The New England Journal of Medicine 2008:9 10.1056/NEJMoa0706467 18172170

[10] Leor J. Sudden Cardiac Death Triggered by an Earthquake. The New England Journal of Medicine 1996;334:7 10.1056/NEJM199601043340102 8552142

[11] Zhou F, Yu T, Du R, et al Clinical course and risk factors for mortality of adult inpatients with COVID-19 in Wuhan, China: a retrospective cohort study. The Lancet 2020:S0140673620305663. 10.1016/S0140-6736(20)30566-3 32171076 PMC7270627

