# Supplementary material for: Hospital admissions during Covid-19 lock-down in Germany: Differences in discretionary and unavoidable cardiovascular events
Source: PLoS One. 2020 Nov 20;15(11):e0242653. doi: 10.1371/journal.pone.0242653 (PMC7678984; doi:10.1371/journal.pone.0242653)
Supplement: S1 Table — (DOCX) [file pone.0242653.s001.docx]

S1 Table. Statistical calculations for Fig 1: March 15- April 30

| Type of admission | Exp. (estimate) | 95% CI lower | 95% CI upper | p-value | adjusted p-value |
| --- | --- | --- | --- | --- | --- |
| Unavoidable | 0.99 | 0.83 | 1.19 | 0.927 | 1 |
| Discretionary | **0.77** | **0.69** | **0.85** | **<0.001** | **<0.001** |
