# Supplementary material for: Hospital admissions during Covid-19 lock-down in Germany: Differences in discretionary and unavoidable cardiovascular events
Source: PLoS One. 2020 Nov 20;15(11):e0242653. doi: 10.1371/journal.pone.0242653 (PMC7678984; doi:10.1371/journal.pone.0242653)
Supplement: S2 Table — (DOCX) [file pone.0242653.s002.docx]

Supplementary Table 2 – Statistical calculations for Fig 2: March 15- April 30

| Type of admission | Exp. (estimate) | 95% CI lower | 95% CI upper | p-value | adjusted p-value |
| --- | --- | --- | --- | --- | --- |
| STEMI | 0.98 | 0.67 | 1.44 | 0.922 | 1 |
| CPR | 0.83 | 0.54 | 1.27 | 0.389 | 1 |
| Stroke | 1.00 | 0.80 | 1.26 | 1.000 | 1 |
| Unstable angina | 0.77 | 0.64 | 0.92 | **0.004** | **0.032** |
| HF | 0.61 | 0.45 | 0.83 | **0.002** | **0.015** |
| COPD | 0.73 | 0.55 | 0.97 | **0.033** | 0.295 |
| Hypertension | 0.85 | 0.69 | 1.05 | 0.138 | 1 |
| Arrhythmia | 0.93 | 0.72 | 1.19 | 0.564 | 1 |
| Dizziness/Syncope | 0.49 | 0.34 | 0.70 | **<0.001** | **<0.001** |
