# Supplementary material for: Hospital admissions during Covid-19 lock-down in Germany: Differences in discretionary and unavoidable cardiovascular events
Source: PLoS One. 2020 Nov 20;15(11):e0242653. doi: 10.1371/journal.pone.0242653 (PMC7678984; doi:10.1371/journal.pone.0242653)
Supplement: S3 Table — (DOCX) [file pone.0242653.s003.docx]

Supplementary Table 3 – Statistical calculations for Fig 3: March 15- April 30

| Category | Exp. (estimate) | 95% CI lower | 95% CI upper | p-value | adjusted p-value |
| --- | --- | --- | --- | --- | --- |
| Female | 0.84 | 0.73 | 0.95 | **0.006** | **0.037** |
| Male | 0.82 | 0.72 | 0.94 | **0.003** | **0.019** |
| Age <60 | 0.94 | 0.77 | 1.13 | 0.496 | 1.000 |
| Age ≥60 | 0.80 | 0.72 | 0.89 | **<0.001** | **<0.001** |
| Suburban | 0.84 | 0.76 | 0.93 | **0.001** | **0.004** |
| Urban | 0.75 | 0.59 | 0.94 | **0.015** | 0.089 |
